# Mitochondrial Gene Expression Profiles Are Associated with Maternal Psychosocial Stress in Pregnancy and Infant Temperament

**DOI:** 10.1371/journal.pone.0138929

**Published:** 2015-09-29

**Authors:** Luca Lambertini, Jia Chen, Yoko Nomura

**Affiliations:** 1 Department of Preventive Medicine, Icahn School of Medicine at Mount Sinai, New York, New York, United States of America; 2 Department of Obstetrics, Gynecology and Reproductive Science, Icahn School of Medicine at Mount Sinai, New York, New York, United States of America; 3 Department of Pediatrics, Icahn School of Medicine at Mount Sinai, New York, New York, United States of America; 4 Department of Oncological Sciences, Icahn School of Medicine at Mount Sinai, New York, New York, United States of America; 5 Department of Psychology, Queens College, CUNY, Flushing, New York, United States of America; 6 Department of Psychiatry, Icahn School of Medicine at Mount Sinai, New York, New York, United States of America; University of Sevilla, SPAIN

## Abstract

**Background:**

Gene-environment interactions mediate through the placenta and shape the fetal brain development. Between the environmental determinants of the fetal brain, maternal psychosocial stress in pregnancy has been shown to negatively influence the infant temperament development. This in turn may have adverse consequences on the infant neurodevelopment extending throughout the entire life-span. However little is known about the underlying biological mechanisms of the effects of maternal psychosocial stress in pregnancy on infant temperament. Environmental stressors such as maternal psychosocial stress in pregnancy activate the stress response cascade that in turn drives the increase in the cellular energy demand of vital organs with high metabolic rates such as, in pregnancy, the placenta. Key players of the stress response cascade are the mitochondria.

**Results:**

Here, we tested the expression of all 13 protein-coding genes encoded by the mitochondria in 108 placenta samples from the Stress in Pregnancy birth cohort, a study that aims at determining the influence of in utero exposure to maternal psychosocial stress in pregnancy on infant temperament. We showed that the expression of the protein-coding mitochondrial-encoded gene *MT-ND2* was positively associated with indices of maternal psychosocial stress in pregnancy including Prenatal Perceived Stress (β = 0.259; p-regression = 0.004; r^2^-regression = 0.120), State Anxiety (β = 0.218; p-regression = 0.003; r^2^-regression = 0.153), Trait Anxiety (β = 0.262; p-regression = 0.003; r^2^-regression = 0.129) and Pregnancy Anxiety Total (β = 0.208; p-regression = 0.010; r^2^-regression = 0.103). In the meantime *MT-ND2* was negatively associated with the infant temperament indices of Activity Level (β = -0.257; p-regression = 0.008; r^2^-regression = 0.165) and Smile and Laughter (β = -0.286; p-regression = 0.036; r^2^-regression = 0.082). Additionally, *MT-ND6* was associated with the maternal psychosocial stress in pregnancy index of Prenatal Perceived Stress (β = -0.231; p-regression = 0.004; r^2^-regression = 0.120), while *MT-CO2* was associated with the maternal psychosocial stress in pregnancy indices of State Anxiety (β = 0.206; p-regression = 0.003; r^2^-regression = 0.153) and Trait Anxiety (β = 0.205; p-regression = 0.003; r^2^-regression = 0.129).

**Conclusions:**

Our data support the role of mitochondria in responding to maternal psychosocial stress in pregnancy, as assessed in placenta, while also suggesting an important role for the mitochondria in the infant temperament development.

## Introduction

Gene-environment interactions are considered more powerful in determining the phenotype as they occur at earlier stages of development triggering both the increase in incidence and the earlier onset of many developmental disorders, including infant temperament disorders [1, 2, 3]. The period of intrauterine development is probably the most critical that can influence infant temperament and neurodevelopment affecting the offspring throughout the entire life [4, 5, 6, 7, 8].

Maternal psychosocial stress in pregnancy (MPSP) is considered a powerful environmental determinant of the infant temperament [9, 10, 11], however little is known on the mechanistic basis of this phenomenon.

MPSP, like any other stress stimulus, is responsible for activating the cascade of events leading to increase in the cellular energy demand and altered calcium (Ca^2+^) homeostasis [12, 13]. In the brain, psychosocial stress increases energy demand by a mechanism referred to as "cerebral insulin suppression". This mechanism limits glucose flux into peripheral tissue to enhance cerebral glucose supply thus affecting the metabolism of many other organs as, particularly, those with high metabolic rates [14, 15, 16, 17].

Mitochondria play a key role in the stress response [13, 18]. Mitochondria act by both providing the cellular energy needed for the stress response by means of the oxidative phosphorylation (OXPHOS) machinery [12] and by actively controlling Ca^2+^ homeostasis, in cooperation with the endoplasmic reticulum [19, 20], thermogenesis, reactive oxygen species generation and apoptosis among others. [21]. Mitochondria are thus among the first responders to stressors [22]. Consequently, modifications of the mitochondrial activity are considered as key events in both acute and chronic homeostatic imbalance [23]. At the same time many neurodevelopmental disorders have been linked to modifications of the mitochondrial activity [21].

Mitochondria are often referred to as the “powerhouse of the cell”, as they produce over 80% of the energy needed to carry out housekeeping and specialized cellular functions [24]. The role of mitochondria is thus more important in vital organs with high metabolic rates and peculiar homeostatic requirements like the brain and muscles and, in pregnancy, the placenta [25, 26].

The placenta is a very dynamic organ with high metabolic rate and tightly regulated homeostatic requirements, where a continuum of phenotypic and morphological changes takes place over the course of gestation [27]. Placental oxygen consumption is second only to that of the fetal brain [28]. By 22 weeks of gestation, the mean placental weight is one third of that of the fetus while the mid-gestation fetus consumes only 25% of the oxygen required by the placenta [29]. The placenta has also been shown, like for the brain, to mainly use glucose to support its high pace metabolism [30, 31]. The placenta acts like the effector of the intrauterine environment to drive embryonic development [32, 33].

Concomitantly, different studies have shown that the fetal brain development is deeply intertwined with that of the placenta [27, 34, 35, 36] that support serotoninergic neuron differentiation by providing serotonin to the developing brain up to the fourteenth week of gestation [37, 38, 39]. Serotonin plays a major role in a variety of cognitive functions [38, 39] and participate in the developmental determination of psychological traits including an increased impulsivity, lower levels of response to inhibition and sensation seeking in toddlers [40].

The placenta also shares the genetic and epigenetic profile of the developing fetus as it originates from the extraembryonic cell layer of the blastocyst [41]. This status affords a unique opportunity of exploring the association of the mitochondria gene expression profile with fetal growth and development by using placental tissue.

In this study we showed that MPSP modifies the mitochondrial gene expression profile in the placenta. We also showed that modifications of placental mitochondria gene expression profiles correlate with the infant temperament development. We conducted our investigation using placenta samples collected by the Stress in Pregnancy (SIP) Study, a birth cohort generated in the New York City metropolitan area that aims at investigating the influence of MPSP on infant temperament.

## Material and Methods

### Study Population

The SIP Study enrolls subjects at Icahn School of Medicine at Mount Sinai, New York Hospital of Queens and Queens College. The SIP Study examines the influence of in utero exposure to MPSP on infant temperament.

MPSP is determined through self-administered questionnaires [42, 43, 44, 45] at the 2^nd^ and 3^rd^ trimesters. MPSP questionnaires are aimed at assessing: objective and subjective levels of stressful experiences, feelings and thoughts during pregnancy, perceived stress, prenatal and perinatal post-traumatic stress symptoms, depressive symptoms and stressful life events.

Questionnaires include: 1) the Perceived Stress Scale (PSS-14) [46], a well-validated 14-item scale instrument that asks about the mother’s feelings and thoughts during the last month (during pregnancy); 2) the Pregnancy Related Anxieties Questionnaire (PRAQ)-Revised [44, 47] which measures five common pregnancy fears subscales (giving birth, bearing a physically or mentally handicapped child, changes and disillusion in partner relationship, changes and concerns about one's mental well-being and the mother-child relationship); 3) the Perinatal PTSD Questionnaire (PPQ) [42, 43] that, with 14 items, measures the severity of maternal PTSD symptoms during prenatal and perinatal periods; and 4) the State-Trait Anxiety Inventory for Adults (STAI) [45] that, with 40 items, measures the temporary condition of “State Anxiety” and long-standing quality of “Trait Anxiety”.

Parental psychopathology is assessed using three detection strategies: 1) direct observation by professionals using the Structured Clinical Interview for DSM-IV Axis I Disorders (SCID-I), used to determine DSM-IV Axis I disorders (major mental disorders) [48]; 2) clinical chart analysis; and 3) self-report by using the Family History Screening [49].

Infant temperament at 6 months of age is assessed by the Infant Behavior Questionnaire Revised (IBQ-R) administered to mothers [50, 51]. IBQ-R items were rationally derived from six constructs (Activity Level, Smile and Laughter, Fear, Distress to Limitations, Duration of Orientation, and Soothability) assessed by aggregating individual items across a range of contexts [52]. The 191 items on the IBQ-R ask parents to rate the frequency of specific temperament-related behaviors observed over the past week. In completing the IBQ-R, parents are asked to read each description of the infant’s behavior, indicating how often the infant engaged in various behaviors during the last week using a 7-point, Likert-type scale for frequency from never to always.

IBQ-R is validated for ages between 3 and 12 months. However it is generally accepted that reliably assessing infant temperament at younger ages is harder than at older ages. Moreover, the timing of the birth (gestational age at birth) could influence the maturity of the infant at earlier ages than older ages. Thus the SIP Study determined that 6 months of age is probably the earliest age for obtaining a reliable assessment.

Importantly, both the MPSP and infant temperament indices return continuous measurements, thus affording the chance of using linear, more informative, models for the analysis of the association of the expression profiles of the protein-coding mitochondrial-encoded genes with both MPSP and infant temperament outcomes.

For our study, summary scores (indices) elaborated from the MPSP and infant temperament questionnaires were used to test their association with the mitochondrial gene expression.

SIP also collects placental tissue at delivery from consented subjects. For this study, placenta samples from 108 subjects enrolled that successfully carried their pregnancies to term were used for the gene expression analysis. All subjects were consented as per the protocol approved by the Institutional Review Boards at Icahn School of Medicine at Mount Sinai, New York Hospital of Queens and Queens College. Informed written consent was obtained from the participants.

### Placental Tissue Collection and RNA Isolation

Placentas were sampled by excising one full-thickness cylindrically shaped biopsy from each of the 4 placenta quadrants midway from the cord insertion and the placental rim, within 2 h from the delivery. Biopsies were initially processed by removing the maternal decidua and fetal membranes and abundantly washing the tissue in cold (4°C) sterile phosphate buffered saline (PBS). Biopsies were then blotted dry, snap-frozen in liquid nitrogen and store at -80°C.

RNA extraction was carried out by first grinding frozen tissue in liquid nitrogen-cooled mortar. Pulverized tissue was then processed for RNA extraction with RNeasy Plus Minikit (Qiagen—Valencia, CA, USA) and quantified with Nanodrop spectrophotometer (Thermo Electron North America—WI, USA). Aliquots of 1 μg of the total RNA extracted were converted into cDNA by using the iScript cDNA Synthesis Kit (BioRad—Hercules, CA, USA) for expression analysis following the manufacturer’s instructions. The rest of the sample was stored at -80°C. The iScript cDNA Synthesis Kit uses a random hexamer primer system to reliably amplify all transcripts in the extracted RNA.

### Mitochondrial Gene Expression Analysis

We tested the expression of the 13 protein-coding mitochondrial-encoded genes listed in S1 Table. Primer sets for the gene expression experiment were designed and validated by: 1) BLASTing (www.blast.ncbi.nlm.nih.gov) the primers sequences against the whole human genome which allowed to determine that our primers did not match any RNA sequence listed by the “refseq” (www.ncbi.nlm.nih.gov/refseq/), “GenBank” (www.ncbi.nlm.nih.gov/genbank/) or UCSC (genome.ucsc.edu/cgi-bin/hgGateway) databases other that the specific mitochondrial genes; 2) testing by electrophoresis in agarose gel that the amplicons generated by the cDNA amplification did not show smears and that their length was within the expected size thus supporting the absence of spurious amplifications of different targets; and 3) verifying the consistency of the melting curves generate per each gene by the light-cycler across the 108 samples tested (see S1 Table for the complete list of primers and amplicons’ length). Primers were designed such to all properly work at the annealing temperature of 63°C in so allowing for thorough randomization of both samples and genes in each real-time PCR plate.

Gene expression was measured by standard real-time PCR in Roche 480 light cycler (Roche Diagnostics—Indianapolis, IN, USA). Cycling conditions for all genes were: 95.0°C for 1 min, followed by 35 cycles of 95.0°C for 30 sec, 63.0°C for 15 sec and 72.0°C for 30 sec. All reactions were run in triplicate and repeated if the standard deviation between the triplicate values was found >1 cycle. Expression values were normalized by first calculating the medians for the expression values of the 13 genes tested per each subject. The median of the subject medians was then calculated and the correction value was determined by subtracting the subject median to median of subject medians. The subject-specific correction value was then applied to each gene.

The expression of the housekeeping gene *ACTB* was used to validate the mitochondrial expression data by verifying that the variations observed in the mitochondrial-encoded gene expression was not due to technical artifacts. The lack of mitochondrial housekeeping genes together with the completely different nature of the transcription process carried out by the nuclear DNA and the mitochondrial DNA in fact warranted the use of a nuclear validated housekeeping gene to verify that the expression variation observed across the mitochondrial-encoded genes was not due to real-time PCR inter-plate variability.

### Statistical Analysis

Statistical modeling was conducted by using stepwise multinomial linear and logistic regression models for, respectively, continuous and nominal outcomes. Normalized gene expression values were used as predictors while controlling for maternal ethnicity, age, marital status, education and welfare, delivery method, gestational age and infant gender. Standardized β values for the expression of mitochondrial-encoded genes significantly associated to the outcomes tested within logistic regression were obtained by applying the “Standardized Coefficients in Logistic Regression” method [53].

The component analysis was carried out by using hierarchical clustering and principal component analysis (PCA). Hierarchical clustering analysis on gene expression values was conducted by Ward Linkage to determine specific gene expression clusters. Dimensional stress analysis of the dataset to validate the clustering analysis was conducted by using multidimensional scaling (MDS). PCA was used to calculate summary scores for each of the expression cluster. PCA was also used to calculate summary scores among MPSP and infant temperament indices as well as infant birth measures.

The following non-parametric tests were used at different stages in the analysis:

1) Spearman's rho for bivariate correlation; 2) Wilcoxon rank-sum to compare mitochondrial gene expression between tertiles of significant MPSP and infant temperament indices; and 3) Kruskal-Wallis to determine the p-trend for mitochondrial gene expression across tertiles of significant MPSP and infant temperament indices.

We used PASW statistical software (version 20) (SPSS Inc.–Chicago, IL, USA) to carry out the statistical analysis.

## Results

### Mitochondrial Gene Expression in the SIP Study Birth Cohort

The population demographics and the characteristics of the variables analyzed are presented in Tables 1 and 2. The population in this study is a subset of the SIP Study birth cohort, a general description of which has been provided in the material and methods section. Briefly, the SIP Study aims at determining the influence of in utero exposure to MPSP on infant temperament.

**Table 1:** Population demographics and variables statistics: nominal.

Variable	N.	%	Variable	N.	%
Mother			*Obsessive Compulsive Disorder*		
*Ethnicity*			No	86	79.6
Latina	54	50.0	Yes	09	08.3
Black	35	32.4	Not Available	13	12.1
Other^(1)^	19	17.6	*Overweight*		
*Marital Status*			No	57	52.8
Married/Common Law	29	26.9	Yes	51	47.2
Single/Divorced/Separated/Widowed	79	73.1	*Obesity*		
*Education*			No	79	73.1
Primary School	36	33.3	Yes	29	26.9
High School	18	16.7	*Delivery Method*		
Some College	36	33.3	Vaginal	73	67.6
BA/Graduate Degree	18	16.7	C-Section	35	32.4
*Welfare Status*			Offspring		
No	16	14.8	*Gender*		
Yes	92	85.2	Male	60	55.6
			Female	48	44.4

^(1)^: Caucasian (white): N = 5 –% = 4.6.

**Table 2:** Population demographics and variables statistics: continuous.

Variable	N.	Mean	St. Dev.	Min	Max
Mother					
Maternal Age (Years)	108	27.36	5.83	17	44
*MPSP Indices*					
Prenatal Perceived Stress	108	37.05	7.33	23	56
State Anxiety	108	38.51	12.15	20	72
Trait Anxiety	108	38.73	11.08	20	64
Pregnancy Anxiety Total	108	6.06	2.33	3.00	12.83
Offspring					
*Infant Birth Measures and Temperament Indices*					
Gestational Age at Birth (Days)	108	274.26	17.16	167	291
Birth Weight (g)	108	3,217.09	630.32	560	4,450
Birth Length (cm)	108	49.80	3.69	29	57
Head Circumference (cm)	108	33.61	2.67	20.5	41.0
Activity Level	67	4.26	1.30	1.86	7.00
Smile and Laughter	67	5.48	1.48	1.14	7.00
*Mitochondrial Gene Expression (Ct Value)*					
*MT-ND1*	108	15.05	2.00	14.79	29.84
*MT-ND2*	108	17.64	2.27	14.35	30.24
*MT-CO1*	108	17.16	2.93	14.04	32.52
*MT-CO2*	108	16.52	2.13	13.03	29.95
*MT-ATP8*	108	16.16	2.25	12.91	27.09
*MT-ATP6*	108	17.50	2.78	13.94	27.56
*MT-CO3*	108	17.68	2.48	14.21	28.10
*MT-ND3*	108	17.23	1.83	14.33	22.26
*MT-ND4L*	108	19.38	3.37	14.10	30.03
*MT-ND4*	108	18.62	3.43	14.90	28.37
*MT-ND5*	108	18.72	2.76	14.50	24.06
*MT-ND6*	108	17.06	2.16	13.70	21.25
*MT-CYB*	108	17.54	2.36	14.61	24.08

Data on MPSP indices and other variables were available across the whole cohort of 108 participants while data on the infant temperament indices were available on only 67 subjects. Not all newborns from the enrolled participants in fact reached age 6 months by the time the study was conducted and infant temperament data were not yet available on all infants.

We quantified the expression of all 13 protein-coding mitochondrial-encoded genes in 108 placenta samples from the SIP Study birth cohort and analyzed their association with MPSP and infant temperament indices. We also investigated additional outcomes, such as maternal psychopathology, maternal weight and infant birth measures, with known association with both high MPSP and pathologic infant temperament phenotypes.

By using multinomial linear and logistic regressions, significant associations with the gene expression were determined for: 1) the MPSP indices of Prenatal Perceived Stress, State Anxiety, Trait Anxiety and Pregnancy Anxiety Total; 2) the infant temperament indices of Activity Level and Smile and Laughter; 3) both maternal overweight and obesity; 4) all infant birth measures including birth weight, birth length and head circumference; and 5) the maternal psychopathology diagnosis of maternal obsessive compulsive disorder.

Significant associations between the mitochondrial gene expression and the MPSP and infant temperament indices listed above were found for genes *MT-ND2*, *MT-ND6* and *MT-CO2* (Table 3). *MT-ND2*, an OXPHOS Complex I gene was found positively associated with all significant MPSP indices and negatively with the infant temperament indices. Another OXPHOS Complex I gene, *MT-ND6*, was found negatively associated with the MPSP index of Prenatal Perceived Stress. *MT-CO2*, an OXPHOS Complex IV gene, was found positively associated with the MPSP indices of State and Trait Anxiety and with maternal overweight.

**Table 3:** Multinomial regression statistics for the correlation between mitochondrial gene expression and each of the analyzed outcomes from SIP.

Outcome		Multinomial Regression				Mitochondrial Genes	
Area	Index	Type	p-value	r^2^	Adj r^2^	Item	β
MPSP	Prenatal Perceived Stress	Linear	**0.004**	0.120	0.100	*MT-ND2*	0.259
						*MT-ND6*	-0.231
	State anxiety	Linear	**0.003**	0.153	0.123	*MT-ND2*	0.218
						*MT-CO2*	0.206
	Trait anxiety	Linear	**0.003**	0.129	0.109	*MT-ND2*	0.262
						*MT-CO2*	0.205
	Pregnancy Anxiety Total	Linear	**0.010**	0.103	0.082	*MT-ND2*	0.208
Psychopath	Obsessive Compulsive Disorder	Logistic	**0.022**	0.135	-	*MT-CO3*	0.048
Maternal Weight	Overweight	Logistic	**0.001**	0.191	-	*MT-CO2*	0.162
						*MT-ND6*	-0.145
	Obesity	Logistic	**<0.001**	0.320	-	*MT-ND1*	0.131
						*MT-ATP6*	0.103
Birth Measures	Birth Weight	Linear	**<0.001**	0.546	0.519	*MT-CO3*	-0.164
	Birth Length	Linear	**<0.001**	0.597	0.583	*MT-ND5*	-0.192
	Head Circumference	Linear	**<0.001**	0.367	0.337	*MT-ND5*	-0.187
						*MT-ATP8*	0.183
Infant	Activity Level	Linear	**0.008**	0.165	0.134	*MT-ND2*	-0.257
Temperament	Smile and Laughter	Linear	**0.036**	0.082	0.064	*MT-ND2*	-0.286

Notes:
Regression p-values are reported as follows: 1) bold & underlined p < 0.01; 2) bold p < 0.05 For the logistic regressions the Nagelkerke pseudo-r^2^ is reported Standardized β values for the logistic regressions have been calculated by using the “Standardized Coefficients in Logistic Regression” method [53].

The expression of other 5 protein-coding mitochondrial-encoded genes, *MT-ND1*, *MT-ND5*, *MT-CO3*, *MT-ATP6*, *MT-ATP8*, was found associated with the maternal psychopathology diagnosis of maternal obsessive compulsive disorder, maternal weight and with infant birth measures (Table 3).

**Fig 1:**
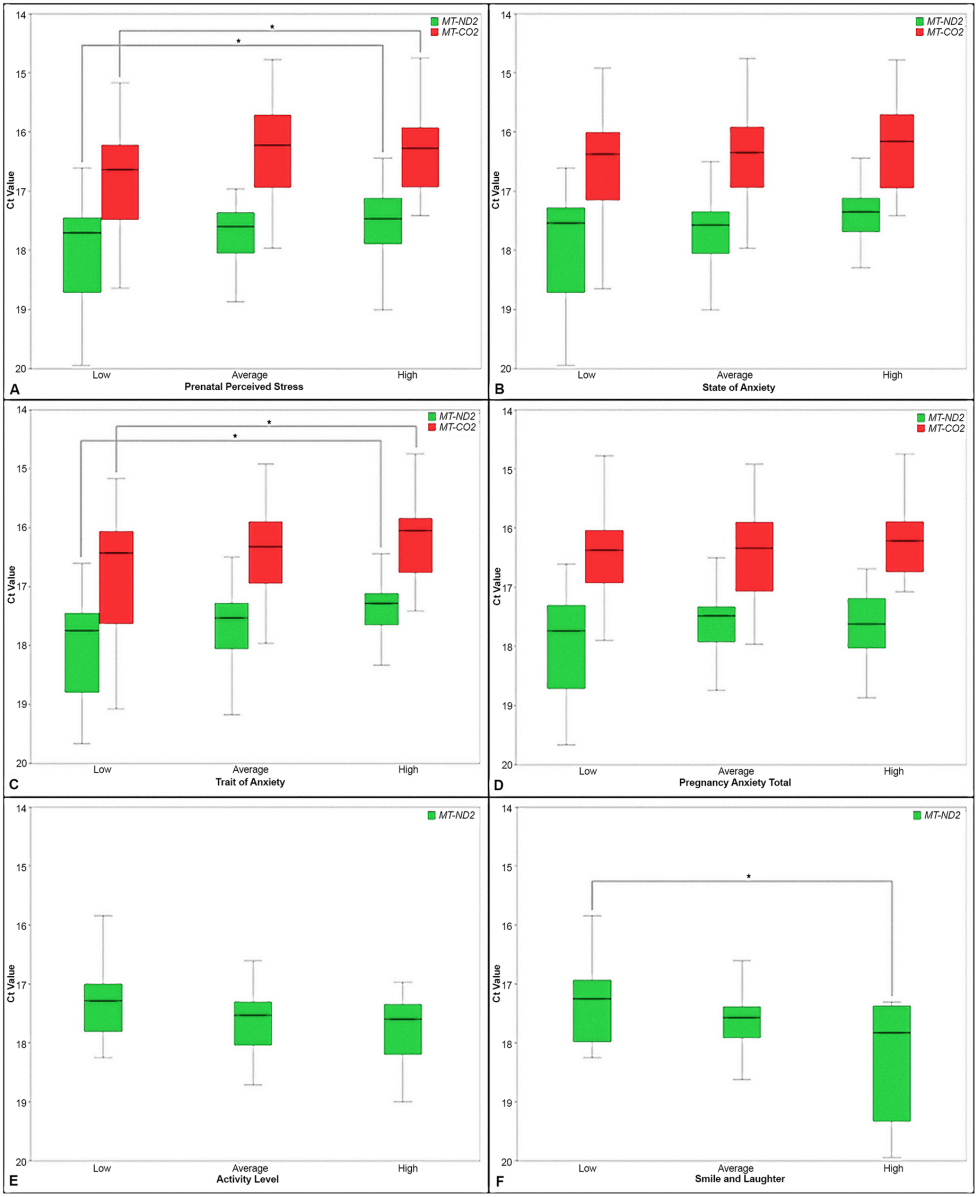
Boxplots of the distribution of the expression of MT-ND2 and MT-CO2 across tertiles of MPSP and infant temperament indices. A-D. MPSP indices. E-F. Infant temperament indices. In each graph “low”, “average” and “high” represent the tertiles of MPSP and infant temperament indices. The star symbol and the bracketing lines represent the tertiles for which significant (p < 0.05) differences in gene expression have been detected.

We further analyzed the association of the placental expression profiles of *MT-ND2* and *MT-CO2* with the significant MPSP indices of Table 3 by categorizing the MPSP indices into tertiles (Fig 1A–1D). Similarly we analyzed the placental expression profiles of *MT-ND2* across tertiles of infant temperament indices (Fig 1E and 1F). *MT-ND6* was not included in this analysis as it was found consistently non-significantly distributed across tertiles of MPSP indices.

Significant upregulation of the gene expression was determined between the low (tertile) and high (tertile) of the MPSP indices of Prenatal Perceived Stress and Trait Anxiety for both *MT-ND2* (p = 0.016 for Prenatal Perceived Stress and p = 0.019 for Trait Anxiety) and *MT-CO2* (p = 0.042 for Prenatal Perceived Stress and p = 0.039 for Trait Anxiety). Overall the expression of both *MT-ND2* and *MT-CO2* showed a consistent trend toward an upregulation across tertiles of MPSP indices (Fig 1A–1D). Trend analysis was also conducted to determine the significance of the expression upregulation of *MT-ND2* and *MT-CO2* across tertiles of MPSP indices. Significant p-values were determined only for *MT-ND2* relatively to the Prenatal Perceive Stress (p = 0.017) and State Anxiety (p = 0.031). Borderline significant p-values were determined for *MT-ND2* for the other MPSP indices as, respectively, Trait Anxiety (p = 0.068) and Pregnancy Anxiety Total (p = 0.059).

*MT-ND2* on the other hand showed a consistent trend to dowregulation across tertiles of infant temperament indices (Fig 1E and 1F). Statistically significant dowregulation of *MT-ND2* was determined between the low (tertile) and high (tertile) of the Smile and Laughter index (Fig 1E and 1F). A significant p-value for downregulation of *MT-ND2* expression across infant temperament tertiles was determined for only Smile and Laughter (p = 0.048).

### Component Analyses

**Fig 2:**
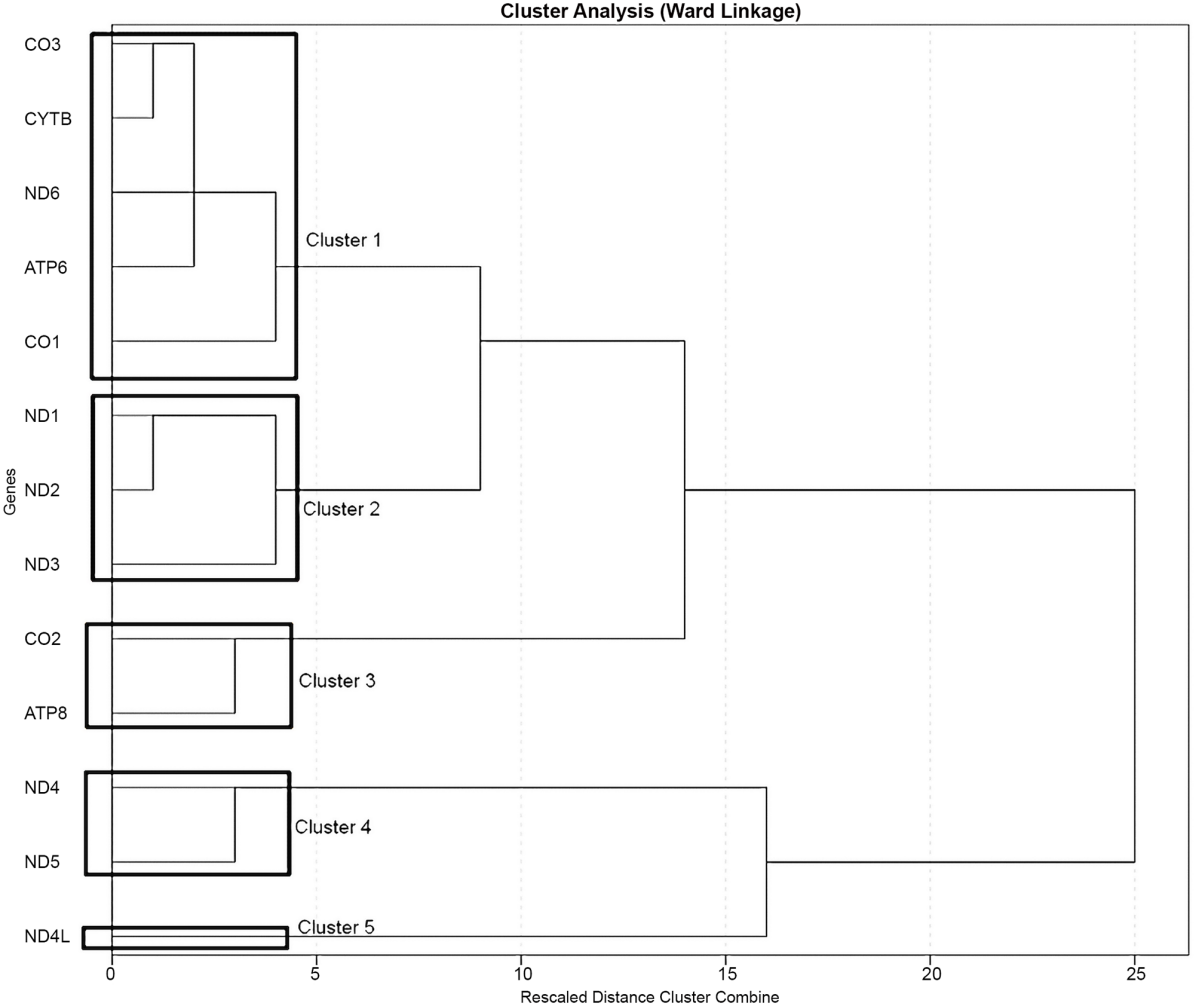
Mitochondrial Expression Clusters. The graph represents the five expression clusters identified for the expression of the 13 protein-coding mitochondrial-encoded genes. The expression clusters have been determined by Ward Linkage. The cutoff level used to generate the clusters in the clustering tree have been chosen accordingly to the multidimensional scaling analysis (see S1 Fig) that showed that the expression data can efficiently be fit into a 5-dimensional space without imposing an excessive degree of stress to the dataset.

As suggested by the role that protein-coding mitochondrial-encoded genes play in the OXPHOS, the expression profiles of the mitochondrial-encoded genes tested showed, for several genes, a relevant degree of colinearity as determined by the high (rho > |0.4|) and significant (p < 0.01) bivariate correlation between samples (S2 Table). To address the effect of colinearity on the significance of our findings we first conducted a clustering analysis that revealed the existence of 5 expression clusters (Fig 2). The expression clusters were then validated by dimensional stress analysis conducted by using multidimensional scaling showing that the expression of the mitochondrial genes can efficiently be fit into a 5 dimension space (e.g. 5 clusters) without imposing an excessive degree of stress to the dataset (S1 Fig). Finally we calculated a summary score for each expression cluster by using principal component analysis (PCA). The efficacy of this approach was further supported by the low bivariate correlation found between the 5 summary scores (S3 Table). Similarly we calculated PCA summary scores for MPSP and infant temperament indices as well as maternal weight (including overweight and obesity) and infant birth measures (including birth weight, birth length and head circumference).

We finally rerun the regression models using the same covariates as above and the PCA summary scores for each expression cluster in place of the individual gene expression values, as predictors, and the PCA summary scores as outcomes in place of the individual outcome indices/values. The results of this test strongly support the validity of our original approach (Table 4). The PCA summary score for the MPSP indices was found positively associated with expression clusters 2 and 3 which include, respectively, genes *MT-ND2* and *MT-CO2* that were previously found positively associated with the individual MPSP indices. A negative non-significant association was detected between expression cluster 2 and the PCA summary score for the infant temperament indices, partially supporting the negative correlation described between the individual infant temperament indices and the expression of *MT-ND2*.

**Table 4:** Multinomial regression statistics for the correlation between the PCA summary scores for the five mitochondrial gene expression clusters and the PCA summary scores for MPSP and infant temperament indices, maternal weight class and infant birth measures.

Index	Multinomial Regression				Expression Clusters	
	Type	p-value	r^2^	Adj r^2^	Item	β
MPSP	Linear	**0.001**	0.166	0.136	Cluster 2	0.228
					Cluster 3	0.218
Maternal Weight	Logistic	**0.006**	0.169	-		
- Normal vs Overweight					Cluster 2	0.107
- Normal vs Obese					Cluster 2	0.145
Birth Measures	Linear	**<0.001**	0.579	0.559	Cluster 4	-0.198
Infant Temperament	Linear	0.277	0.311	0.064	Cluster 2	-0.278

Notes:
Regression p-values are reported as follows: 1) bold & underlined p < 0.01; 2) bold p < 0.05; 3) regular p ≥ 0.05 For the logistic regressions the Nagelkerke pseudo-r squared is reported Standardized β values for the logistic regressions have been calculated by using the “Standardized Coefficients in Logistic Regression” method [53].

Additionally expression cluster 2 was positively associated with the PCA summary score for maternal weight. Expression cluster 2 includes gene *MT-ND1* which was the gene with the most significant (positive) association with maternal obesity. Expression cluster 4 was instead found negatively associated with the PCA summary score for infant birth measures supporting the association previously found for *MT-ND5*, the only gene associated with two birth measures (birth length and head circumference).

**Fig 3:**
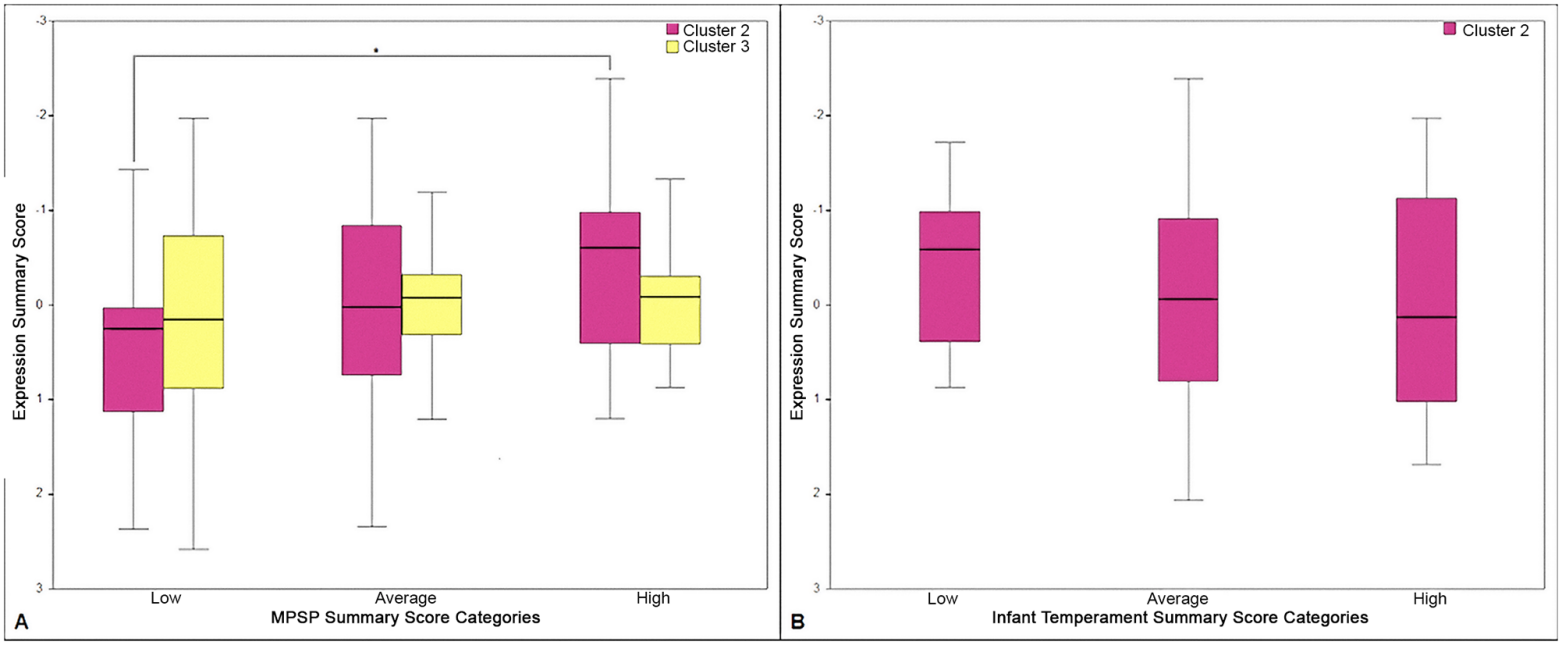
Boxplots representing the distribution of the expression clusters 2 and 3 across tertiles of PCA summary score for MPSP and infant temperament indices. A. PCA summary score for MPSP indices. B. PCA summary score for infant temperament indices. In each graph “low”, “average” and “high” represent the tertiles of the PCA summary score for MPSP and infant temperament indices. The star symbol and the bracketing lines represent the tertiles for which significant (p < 0.05) differences in gene expression have been detected.

In line with our prior analysis, we tested the placental expression profile of expression clusters 2 and 3 across tertiles of the PCA summary score for the MPSP indices and the placental expression profile of expression cluster 2 across PCA summary score for the infant temperament indices (Fig 3). A statistically significant upregulation of the score for the expression cluster 2 was determined between the low (tertile) and the high (tertile) of the PCA summary score for the MPSP indices. This finding indicates that the genes of this expression cluster (see Fig 2 for reference) follow a similar expression trend. These data are partially supported by a borderline significant p-value for trend determined for expression cluster 2 across the tertiles of PCA summary score for the MPSP indices (p = 0.079). Expression cluster 3 showed a similar but less marked and non-significant expression trend (Fig 3A).

A non-significant downregulation of the score for the expression cluster 2 was observed across tertiles of PCA summary score for the infant temperament indices (Fig 3B).

### Mitochondrial Gene Expression and the Expression of Corticotropin and Glucocorticoid Receptors in Placenta

In a previous investigation on a subset of 50 among the 108 samples used here [54], we found limited evidence of the association between the expression of *CRHR1*, *CRHR2* and *NR3C1*, three key hormonal receptor of the HPA (Hypothalamic-Pituitary-Adrenal) axis, and both MPSP and infant temperament indices. The corticotropin-releasing hormone (*CRH*) receptors *CRHR1* and *CRHR2* and the glucocorticoids’ (GHs) receptor *NR3C1* regulate the HPA axis, a fundamental mediator of the stress response and a modulator of the mitochondrial activity [55].

We tested the interaction between the HPA axis and the mitochondrial gene expression in determining the tested outcomes by modeling each outcome within linear and logistic regressions. We used as predictors the same covariates as in the previous analyses together with the PCA summary scores for each mitochondrial expression cluster and the normalized expression values for *CRHR1*, *CRHR2* and *NR3C1*. The only association with the outcomes was found for *CRHR1* that was positively associated with the MPSP index of State Anxiety together, as previously determined, with mitochondrial expression cluster 2 (S4 Table).

Noticeably, we also found a high degree of significant negative bivariate correlation between the expression of *NR3C1* and *MT-CO1* (S5 Table).

## Discussion

In this study we explored the role of the mitochondrial gene expression in responding to MPSP, a powerful environmental determinant of fetal brain development [3, 9, 10, 11], affecting infant temperament. By using placenta samples from the SIP Study birth cohort we determined that the expression of 3 of the 13 protein-coding mitochondrial-encoded genes of the OXPHOS, *MT-ND2*, *MT-ND6*, *MT-CO2*, is correlated to both MPSP and infant temperament indices. The expression of other 5 protein-coding mitochondrial-encoded genes was found associated with maternal and infant outcomes associated with high MPSP and pathologic infant temperament phenotypes.

MPSP predicts considerable portions of the variance in infant neurodevelopment leading to lower mental development scores and irritable temperament [9, 56, 57, 58]. This scenario theoretically confers an evolutionary advantage to the progeny [59, 60, 61] as the increased levels of anxiety in the offspring of mothers experiencing high MPSP may in fact be explained by increased vigilance and alertness to danger [62]. These elevated rates of anxiety, often accompanied by increased aggression, may thus direct a process of adaptation leading to a higher sensibility to adverse environments, higher drive in exploring new environments and fight possible predators and lower proneness to engage in developing deep relationships with other individuals [63, 64, 65].

MPSP indices that were found associated with the expression profiles of mitochondrial genes in this study are representative of objective and subjective levels of stressful experiences, feelings and thoughts during pregnancy and perceived stress [42, 43, 44, 46, 47, 66]. Infant temperament indices significantly associated with the expression profiles of mitochondrial genes belong to the temperament developmental factors of Surgency/Extraversion (Activity Level) and Effortful Control (Smile and Laughter). High scores for indices grouped under these temperament developmental factors are representative of the infant temperament phenotypes of, respectively, anxiety [67, 68, 69] and aggression [70, 71]. While it is beyond the scope of the current study, it is worth to additionally report that influences of postnatal psychosocial rearing environment on infant temperament at 6 months of age, as especially maternal postpartum depression, have been found not to affect infant temperament indices as determined by the IBQ-R in the SIP Study birth cohort (data not shown).

Stress stimuli like MPSP set off the physiologic stress response involving mitochondria [12, 13, 18]. Mitochondria code for 13 proteins of the OXPHOS system [24]. Eleven of these 13 proteins represent the building blocks of 3 OXPHOS protein complexes, the NADH dehydrogenase (Complex I), the cytochrome c reductase (Complex III) and the cytochrome c oxidase (Complex IV), that, together with the entirely nuclear-encoded succinate dehydrogenase (Complex II), form the electron transport chain (ETC). Two additional mitochondrial proteins regulate the activity of the ATP synthase (Complex V).

In our study the expression of OXPHOS Complex I gene *MT-ND2* showed the strongest association with both MPSP and infant temperament indices. Interestingly MPSP, which is known to negatively affect infant temperament, in this study was associated with an increase on the expression of *MT-ND2* that then shows a negative association with the infant temperament indices. *MT-ND6*, another member of the OXPHOS Complex I, shows instead a decreased expression even if limited to the MPSP index of Prenatal Perceived Stress.

OXPHOS Complex I, the initiator of the mitochondrial ETC [24], is a large multiprotein complex arranged in three modules known as N, Q and P. The P module is made out of a proximal and distal portion [72, 73] organized into a transmembrane proton pump that can work uncoupled from the ETC [73]. Genes *MT-ND1*, *MT-ND2*, *MT-ND3* and *MT-ND4L* belong to the proximal portion of the P module while *MT-ND4* and *MT-ND5* belong to the distal portion [72]. *MT-ND6* instead belongs to the P-Q bridging region of the Q module [72, 74]. Interestingly, in our study we found that *MT-ND1*, *MT-ND2* and *MT-ND3* expression profiles group together in expression cluster 2, while *MT-ND4* and *MT-ND5* form expression cluster 4 in so mirroring the P module organization. *MT-ND6* separately clusters with other genes to form expression cluster 1. The parallel between the cluster distribution of Complex I genes and the topology of the P module substantially adds confidence to the results of the clustering analysis.

Complex I alone contributes for 40% to the generation of the mitochondrial proton-motive force utilized for ATP synthesis and transport processes and it is a major contributor to reactive oxygen species production in the cell [75]. Complex I deficiency is also the most frequent defects of mitochondrial energy metabolism [76] which have been linked to a wide range of neurodevelopmental and neurodegenerative disorders [75].

Complex I is thus likely a key player of the response to stressors such as MPSP. Such response entails the activation of the OXPHOS to respond to the increased energy demand [12, 13] for actively regulating the Ca^2+^ cellular homeostasis [12, 13, 37]. Ca^2+^ in turns cumulates in the mitochondria, increases the OXPHOS rate and ATP production and act as a regulator of the secretion of many neurotransmitters such as serotonin [37]. Serotonin regulates neurodevelopmental processes through maternal-fetal interactions that have long-term mental health implications [37]. Importantly, the placenta supplies serotonin to the developing brain to support neuronal differentiation [37].

As the P module evolved from passive bacterial kation antiporter [77], our data are in agreement with the literature reporting a partial uncoupling of the P module of Complex I with the ETC in response to the increased mitochondrial concentration of Ca^2+^ [73, 78]. Stress-driven Ca^2+^ sequestration from the cytoplasm into the mitochondria however results in decreased serotonin release that may alter fetal development [37]. Accordingly *MT-ND2* expression profile was found negatively associated with infant temperament.

The high rates of ATP production in response to the increased energy demand brought about by MPSP may also explain the expression pattern of Complex IV genes. Complex IV is the last component of the ETC catalyzing the conversion of oxygen, the final acceptor of the electrons of the ETC, into water [24]. *MT-CO2* positive expression association with the MPSP indices of State and Trait Anxiety may be linked to a higher OXPHOS activity. The positive correlation between *MT-CO3* expression and maternal obsessive compulsive disorder lend further support to this hypothesis. Maternal obsessive compulsive disorder in our dataset is in fact significantly associated with each of the MPSP indices (S6 Table) in agreement with the existing literature [79, 80, 81, 82].

The high incidence of overweight (~47%) and obesity (~27%) (Table 1) within the SIP population analyzed here add an additional workload on mitochondria. Alterations of the mitochondrial activity have in fact been reported in placentas from women with elevated BMI [83]. Particularly, a large number of studies show that obesity leads to dissipations of the mitochondrial membrane potential [84, 85, 86]. The positive correlation between overweight and *MT-CO2* expression and between obesity and *MT-ND1* and *MT-ATP6* expression that we determined in the SIP cohort placentas can therefore be attributable to the attempt by mitochondria to respond to the MPSP effect while also battling the effect brought about by obesity.

In our investigation we found several examples of increased vs decreased mitochondrial gene expression (e.g. *MT-ND2* and *MT-ND6*) in association with the MPSP and infant temperament indices (Table 3). Accordingly we also determined the existence of significant negative bivariate correlation between the expression profiles of mitochondrial genes (S2 Table). However, mitochondrial genes are transcribed into polycistronic RNAs, one per each mitochondrial DNA strand. These transcripts are then cleaved and processed for translation into proteins. Such transcription method would speak for a coordinated regulation of the expression of all mitochondrial genes returning a homogenous expression profile and high positive correlation scores between genes. However mechanistic studies have shown that the mitochondrial polycistronic RNAs, before translation, undergo differential cleavage/degradation in order to respond to specific physiologic needs that accordingly modulate the expression of only some mitochondrial genes [87]. Additionally several studies reported that the expression of individual mitochondrial genes undergo to differential regulation [18, 88], supporting the findings of our study. These data are also in agreement with the role that mitochondrial-encoded genes play within their OXPHOS complexes. Mitochondrial genes of complexes I, III and IV in fact code for the catalytic components of their respective complexes [72, 89] and their expression profiles are differentially related to each other and unrelated to the chaperon-like nuclear-encoded subunits of the same complexes.

While partial and preliminary, our interpretation is further supported by the integrated analysis with the expression data on *CRH* and GHs receptors (GRs). As expected, *CRH* receptors’ expression was found associated with MPSP indices in conjunction with mitochondrial genes of the P module of Complex I. Concurrently a significant correlation was found between the GRs and the expression of the complex IV gene *MT-CO1*, supporting the findings that showed that GRs participate in the modulation of the mitochondrial activity [55]. Mitochondria in fact carry 4 glucocorticoid response elements within the *MT-CO1* gene [90] and the activated *NR3C1* has been shown to bind all four of them [91].

These data support the role that HPA axis plays in modulating the energy production through hormonal feedback cycles. Our data also suggest that the HPA axis activation may be a more transient phenomenon that participates in determining long lasting changes that reflect in the association between the mitochondrial expression and infant temperament indices and birth measures.

Overall this study highlights the importance of placental mitochondria gene expression in responding to MPSP while suggesting a possible role of placental mitochondria gene expression in affecting infant temperament. Additional data are however needed to better describe the mitochondrial response to stressors like MPSP. The increased sample size of the cohort would be of great benefit to support the validity of these findings in future investigations while also allowing for the study of the role of the placental mitochondria gene expression in mediating/moderating the effects of MPSP on infant temperament. The analysis of the mitochondrial metabolism by means of enzymatic assays targeting Complex I and IV activity rates would also provide important information of the mitochondrial functioning.

## Supporting Information

S1 Fig Multidimensional scaling analysis stress plot. The graph shows that the expression of the mitochondrial genes can efficiently be fit into a 5 dimension space (e.g. 5 clusters) without imposing an excessive degree of stress to the dataset.
(DOCX)

S1 Table List of the mitochondrially-encoded genes, primer sequences and amplicons lengths used in this study. (DOCX)

S2 Table Non-Parametric Bivariate Correlation (Spearman’s rho) between the expression of mitochondrial genes. (DOCX)

S3 Table Non-Parametric Bivariate Correlation (Spearman’s rho) between the Mitochondrial Expression Clusters. (DOCX)

S4 Table Multinomial linear regression for the association of the MPSP index of State anxiety with the cluster of mitochondrial gene expression and the expression of the CRHR1, CRHR2 and NR3C1 hormonal receptors of the HPA axis. (DOCX)

S5 Table Non-Parametric Bivariate Correlation (Spearman’s rho) between the expression of the mitochondrial gene and the expression of *CRHR1*, *CRHR2* and *NR3C1* hormonal receptors of the HPA axis. (DOCX)

S6 Table Distribution of the MPSP individual indices and the MPSP summary score by Maternal Obsessive Compulsive Disorder (OCD). (DOCX)
