# Supplementary material for: Mitochondrial Gene Expression Profiles Are Associated with Maternal Psychosocial Stress in Pregnancy and Infant Temperament
Source: PLoS One. 2015 Sep 29;10(9):e0138929. doi: 10.1371/journal.pone.0138929 (PMC4587925; doi:10.1371/journal.pone.0138929)
Supplement: S1 Fig — The graph shows that the expression of the mitochondrial genes can efficiently be fit into a 5 dimension space (e.g. 5 clusters) without imposing an excessive degree of stress to the dataset. (DOCX) [file pone.0138929.s001.docx]

| 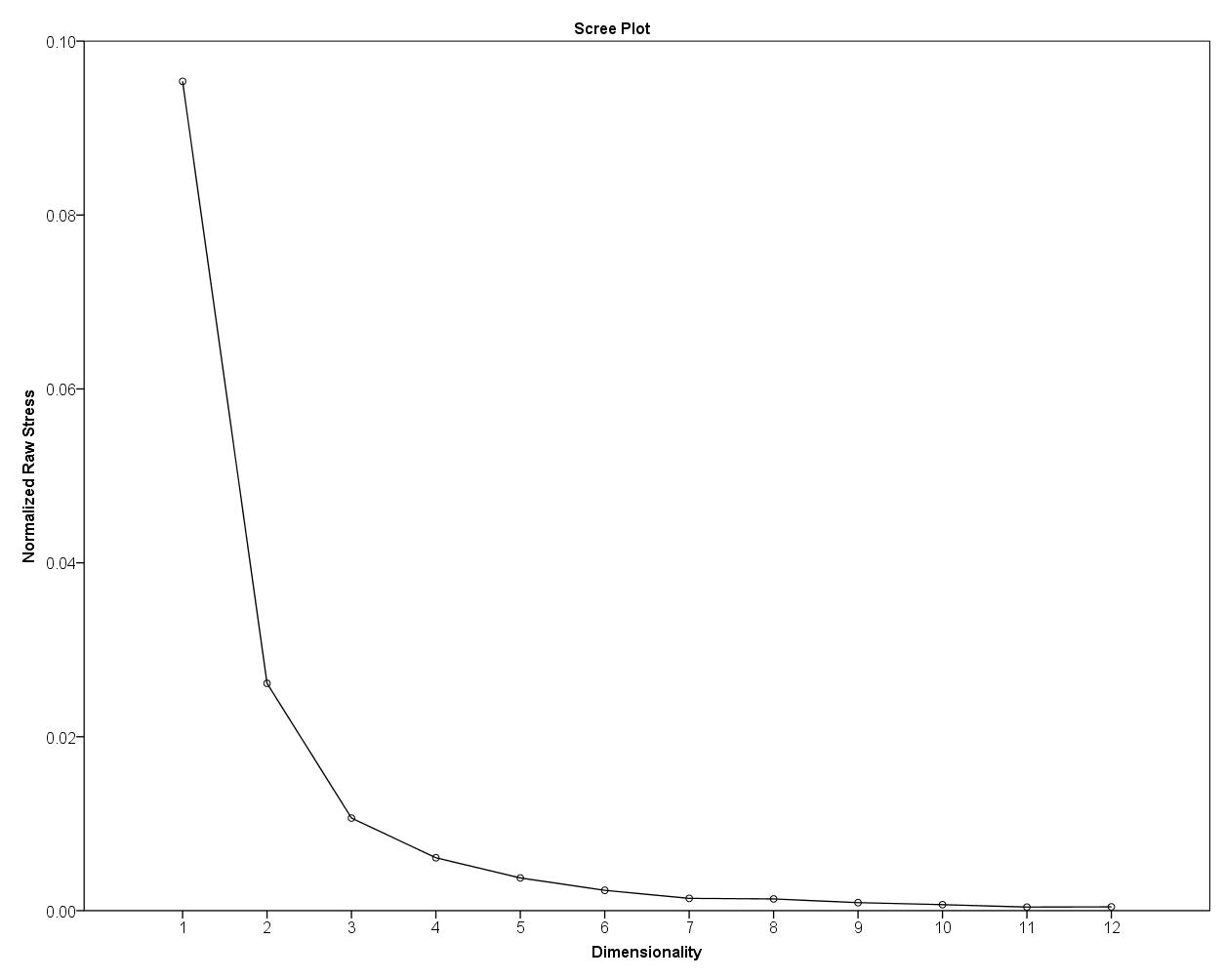 | |
| --- | --- |
| Figure S1. | Multidimensional scaling (MDS) analysis stress plot. The graph shows that the expression of the mitochondrial genes can efficiently be fit into a 5 dimension space (e.g. 5 clusters) without imposing an excessive degree of stress to the dataset. |
