# Supplementary material for: Mitochondrial Gene Expression Profiles Are Associated with Maternal Psychosocial Stress in Pregnancy and Infant Temperament
Source: PLoS One. 2015 Sep 29;10(9):e0138929. doi: 10.1371/journal.pone.0138929 (PMC4587925; doi:10.1371/journal.pone.0138929)
Supplement: S1 Table — (DOCX) [file pone.0138929.s002.docx]

| Table S1. | List of the mitochondrially-encoded genes. | | | | | |
| --- | --- | --- | --- | --- | --- | --- |
| Gene Symbol | Gene Name | OXPHOS Complex | Gene Coordinates | Coding Strand | Primer Sequence | Amplicon Length (bp) |
| *MT-ND1* | NADH-Ubiquinone Oxidoreductase Chain 1 | I | 3,307 – 4,262 | Heavy (+) | FWD: CGCACTCTCCCCTGAACTCT REV: AGTTGGTCGTAGCGGAATCG | 111 |
| *MT-ND2* | NADH-Ubiquinone Oxidoreductase Chain 2 | I | 4,470 – 5,511 | Heavy (+) | FWD: CCCGCTAACCGGCTTTTT REV: GGAGGGTGATGGTGGCTATG | 98 |
| *MT-CO1* | Cytochrome c Oxidase Subunit 1 | IV | 5,904 – 7,445 | Heavy (+) | FWD: AGGGGCCATCAATTTCATCA REV: GGACGGATCAGACGAAGAGG | 89 |
| *MT-CO2* | Cytochrome c Oxidase Subunit 2 | IV | 7,586 – 8,269 | Heavy (+) | FWD: GATTGAAGCCCCCATTCGTA REV: TCCGGGAATTGCATCTGTTT | 97 |
| *MT-ATP8* | ATP Synthase F_0_ Subunit 8 | V | 8,366  – 8,572 | Heavy (+) | FWD: CCCCCATACTCCTTACACTATTCC REV: GGGCTTTGGTGAGGGAGGT | 83 |
| *MT-ATP6* | ATP Synthase F_0_ Subunit 6 | V | 8,527 – 9,207 | Heavy (+) | FWD: GCGGGCGCAGTGATTATAGG REV: ATGGGGATAAGGGGTGTAGG | 92 |
| *MT-CO3* | Cytochrome C Oxidase Subunit 3 | IV | 9,207 – 9,990 | Heavy (+) | FWD: CGGCCTAGCCATGTGATTTC REV: CGCGCCATCATTGGTATATG | 92 |
| *MT-ND3* | NADH-Ubiquinone Oxidoreductase Chain 3 | I | 10,059 – 10,404 | Heavy (+) | FWD: TGCGGCTTCGACCCTATATC REV: AGGGGTAAAAGGAGGGCAAT | 110 |
| *MT-ND4L* | NADH-Ubiquinone Oxidoreductase Chain 4L | I | 10,470 – 10,766 | Heavy (+) | FWD: TCATAACCCTCAACACCCACTC REV: CTAGGCCCACCGCTGCT | 90 |
| *MT-ND4* | NADH-Ubiquinone Oxidoreductase Chain 4 | I | 10,760 – 12,137 | Heavy (+) | FWD: CTTTTCCTCCGACCCCCTAA REV: GGATAAGTGGCGTTGGCTTG | 91 |
| *MT-ND5* | NADH-Ubiquinone Oxidoreductase Chain 5 | I | 12,337 – 14,148 | Heavy (+) | FWD: CCTGACTCCCCTCAGCCATA REV: GGGTGGAAGCGGATGAGTAA | 103 |
| *MT-ND6* | NADH-Ubiquinone Oxidoreductase Chain 6 | I | 14,149 – 14,673 | Light (-) | FWD: ATCAACCCTGACCCCTCTCC REV: TTGGTGCTGTGGGTGAAAGA | 101 |
| *MT-CYB* | Cytochrome b | III | 14,747 – 15,887 | Heavy (+) | FWD: AGACAGTCCCACCCTCACAC REV: GTTGTTTGATCCCGTTTCGT | 112 |
