# Supplementary material for: Mitochondrial Gene Expression Profiles Are Associated with Maternal Psychosocial Stress in Pregnancy and Infant Temperament
Source: PLoS One. 2015 Sep 29;10(9):e0138929. doi: 10.1371/journal.pone.0138929 (PMC4587925; doi:10.1371/journal.pone.0138929)
Supplement: S2 Table — (DOCX) [file pone.0138929.s003.docx]

| Table S2. | | Non-Parametric Bivariate Correlation (Spearman’s rho) between the expression of mitochondrial genes. | | | | | | | | | | | | | |
| --- | --- | --- | --- | --- | --- | --- | --- | --- | --- | --- | --- | --- | --- | --- | --- |
|  | | | *MT-ND1* | *MT-ND2* | *MT-CO1* | *MT-CO2* | *MT-ATP8* | *MT-ATP6* | *MT-CO3* | *MT-ND3* | *MT-ND4L* | *MT-ND4* | *MT-ND5* | *MT-ND6* | *MT-CYB* |
| *MT-ND1* | Correlation Coefficient | | 1.000 | .370 | -.359 | ***.433*** | .099 | -.186 | -.098 | ***.402*** | .014 | -.365 | -.229 | -.329 | .105 |
|  | Sig. (2-tailed) | | – | <.001 | <.001 | ***<.001*** | .334 | .068 | .341 | ***<.001*** | .895 | <.001 | .025 | .001 | .308 |
| *MT-ND2* | Correlation Coefficient | |  | 1.000 | -.169 | .205 | -.110 | -.202 | -.337 | .020 | -.077 | -.319 | -.120 | -.094 | .068 |
|  | Sig. (2-tailed) | |  | – | .102 | .046 | .289 | .049 | .001 | .850 | .461 | .002 | .250 | .366 | .518 |
| *MT-CO1* | Correlation Coefficient | |  |  | 1.000 | -.311 | -.355 | -.069 | .010 | ***-.627*** | -.262 | ***.461*** | .306 | .309 | .003 |
|  | Sig. (2-tailed) | |  |  | – | .002 | <.001 | .501 | .926 | ***<.001*** | .010 | ***<.001*** | .002 | .002 | .977 |
| *MT-CO2* | Correlation Coefficient | |  |  |  | 1.000 | .114 | -.227 | -.043 | ***.411*** | .133 | ***-.445*** | -.315 | ***-.456*** | .019 |
|  | Sig. (2-tailed) | |  |  |  | – | .267 | .026 | .672 | ***<.001*** | .198 | ***<.001*** | .002 | ***<.001*** | .854 |
| *MT-ATP8* | Correlation Coefficient | |  |  |  |  | 1.000 | .112 | .055 | .312 | -.071 | -.136 | .260 | -.027 | ***-.418*** |
|  | Sig. (2-tailed) | |  |  |  |  | – | .249 | .573 | .001 | .470 | .162 | .007 | .780 | ***<.001*** |
| *MT-ATP6* | Correlation Coefficient | |  |  |  |  |  | 1.000 | -.306 | -.279 | -.389 | .368 | .181 | -.182 | -.120 |
|  | Sig. (2-tailed) | |  |  |  |  |  | – | .001 | .004 | <.001 | <.001 | .062 | .061 | .219 |
| *MT-CO3* | Correlation Coefficient | |  |  |  |  |  |  | 1.000 | .201 | .018 | -.133 | .025 | -.030 | -.078 |
|  | Sig. (2-tailed) | |  |  |  |  |  |  | – | .038 | .855 | .173 | .796 | .760 | .424 |
| *MT-ND3* | Correlation Coefficient | |  |  |  |  |  |  |  | 1.000 | .157 | ***-.514*** | -.287 | -.197 | -.035 |
|  | Sig. (2-tailed) | |  |  |  |  |  |  |  | – | .106 | ***<.001*** | .003 | .042 | .718 |
| *MT-ND4L* | Correlation Coefficient | |  |  |  |  |  |  |  |  | 1.000 | -.277 | -.208 | .073 | -.123 |
|  | Sig. (2-tailed) | |  |  |  |  |  |  |  |  | – | .004 | .031 | .458 | .208 |
| *MT-ND4* | Correlation Coefficient | |  |  |  |  |  |  |  |  |  | 1.000 | .164 | -.118 | .119 |
|  | Sig. (2-tailed) | |  |  |  |  |  |  |  |  |  | – | .091 | .226 | .223 |
| *MT-ND5* | Correlation Coefficient | |  |  |  |  |  |  |  |  |  |  | 1.000 | .280 | ***-.550*** |
|  | Sig. (2-tailed) | |  |  |  |  |  |  |  |  |  |  | – | .004 | ***<.001*** |
| *MT-ND6* | Correlation Coefficient | |  |  |  |  |  |  |  |  |  |  |  | 1.000 | -.163 |
|  | Sig. (2-tailed) | |  |  |  |  |  |  |  |  |  |  |  | – | .093 |
| *MT-CYB* | Correlation Coefficient | |  |  |  |  |  |  |  |  |  |  |  |  | 1.000 |
|  | Sig. (2-tailed) | |  |  |  |  |  |  |  |  |  |  |  |  | – |
| Strong (rho > 0.4) and significant (p < 0.05) non-parametric correlations between the expression of individual mitochondrial genes are reported in bold italicized. | | | | | | | | | | | | | | | |
