# Supplementary material for: Mitochondrial Gene Expression Profiles Are Associated with Maternal Psychosocial Stress in Pregnancy and Infant Temperament
Source: PLoS One. 2015 Sep 29;10(9):e0138929. doi: 10.1371/journal.pone.0138929 (PMC4587925; doi:10.1371/journal.pone.0138929)
Supplement: S3 Table — (DOCX) [file pone.0138929.s004.docx]

| Table S3. | | Non-Parametric Bivariate Correlation (Spearman’s rho) between the Mitochondrial Expression Clusters | | | | | |
| --- | --- | --- | --- | --- | --- | --- | --- |
|  | | | Cluster 1 | Cluster 2 | Cluster 3 | Cluster 4 | Cluster 5 |
| Cluster 1 | Correlation Coefficient | | 1.000 | -.230 | -.388 | .062 | -.043 |
|  | Sig. (2-tailed) | | – | .026 | <.001 | .546 | .675 |
| Cluster 2 | Correlation Coefficient | |  | 1.000 | .391 | -.008 | -.005 |
|  | Sig. (2-tailed) | |  | – | <.001 | .939 | .958 |
| Cluster 3 | Correlation Coefficient | |  |  | 1.000 | .070 | -.028 |
|  | Sig. (2-tailed) | |  |  | – | .498 | .785 |
| Cluster 4 | Correlation Coefficient | |  |  |  | 1.000 | -.070 |
|  | Sig. (2-tailed) | |  |  |  | – | .475 |
| Cluster 5 | Correlation Coefficient | |  |  |  |  | 1.000 |
|  | Sig. (2-tailed) | |  |  |  |  | – |
| Strong (rho > 0.4) and significant (p < 0.05) non-parametric correlations between individual cluster of the expression of mitochondrial genes are reported in bold italicized. | | | | | | | |
