# Supplementary material for: Mitochondrial Gene Expression Profiles Are Associated with Maternal Psychosocial Stress in Pregnancy and Infant Temperament
Source: PLoS One. 2015 Sep 29;10(9):e0138929. doi: 10.1371/journal.pone.0138929 (PMC4587925; doi:10.1371/journal.pone.0138929)
Supplement: S4 Table — (DOCX) [file pone.0138929.s005.docx]

| Table S4. | Multinomial linear regression for the association of the MPSP index of State of Anxiety with the cluster of mitochondrial gene expression and the expression of the *CRHR1*, *CRHR2* and *NR3C1* hormonal receptors of the HPA axis. |
| --- | --- |
| \| **Model Summary** \| \| \| \| \| --- \| --- \| --- \| --- \| \| R \| R Square \| Adjusted R Square \| Std. Error of the Estimate \| \| .556^a^ \| .310 \| .267 \| 9.702 \| \| a. Predictors: (Constant), Cluster 2, *CRHR1* \| \| \| \|  \| **ANOVA^a^** \| \| \| \| \| \| --- \| --- \| --- \| --- \| --- \| \|  \| Sum of Squares \| Mean Square \| F \| Sig. \| \| Regression \| 1351.037 \| 675.518 \| 7.177 \| .003^b^ \| \| Residual \| 3011.935 \| 94.123 \|  \|  \| \| Total \| 4362.971 \|  \|  \|  \| \| a. Dependent Variable: State of Anxiety \| \| \| \| \| \| b. Predictors: (Constant), Cluster 2, *CRHR1* \| \| \| \| \|  \| **Coefficients^a^** \| \| \| \| \| \| --- \| --- \| --- \| --- \| --- \| \|  \| Unstandardized Coefficients \| \| Standardized Coefficients \| Sig. \| \| B \| Std. Error \| Beta \| \| (Constant) \| 98.828 \| 26.331 \|  \|  \| \| Cluster 2 \| 7.921 \| 2.792 \| .417 \| .008 \| \| *CRHR1* \| .661 \| .266 \| .365 \| .018 \| \| a. Dependent Variable: State of Anxiety \| \| \| \| \| | |
|  | |
