# Supplementary material for: Mitochondrial Gene Expression Profiles Are Associated with Maternal Psychosocial Stress in Pregnancy and Infant Temperament
Source: PLoS One. 2015 Sep 29;10(9):e0138929. doi: 10.1371/journal.pone.0138929 (PMC4587925; doi:10.1371/journal.pone.0138929)
Supplement: S5 Table — (DOCX) [file pone.0138929.s006.docx]

| Table S5. | Non-Parametric Bivariate Correlation (Spearman’s rho) between the expression of the mitochondrial gene and the expression of *CRHR1*, *CRHR2* and *NR3C1* hormonal receptors of the HPA axis | | | | |
| --- | --- | --- | --- | --- | --- |
|  | | | *CRHR1* | *CRHR2* | *NR3C1* |
| *MT-ND1* | | Correlation Coefficient | -.144 | -.054 | -.119 |
|  |  | Sig. (2-tailed) | .395 | .754 | .463 |
| *MT-ND2* | | Correlation Coefficient | .056 | .061 | .042 |
|  |  | Sig. (2-tailed) | .748 | .726 | .804 |
| *MT-CO1* | | Correlation Coefficient | -.110 | -.161 | ***-.425*** |
|  |  | Sig. (2-tailed) | .515 | .349 | ***.006*** |
| *MT-CO2* | | Correlation Coefficient | -.075 | -.310 | .069 |
|  |  | Sig. (2-tailed) | .659 | .066 | .672 |
| *MT-ATP8* | | Correlation Coefficient | .036 | -.109 | -.038 |
|  |  | Sig. (2-tailed) | .813 | .483 | .796 |
| *MT-ATP6* | | Correlation Coefficient | .043 | -.192 | .011 |
|  |  | Sig. (2-tailed) | .777 | .211 | .940 |
| *MT-CO3* | | Correlation Coefficient | .205 | .185 | .134 |
|  |  | Sig. (2-tailed) | .177 | .229 | .359 |
| *MT-ND3* | | Correlation Coefficient | .176 | -.036 | .122 |
|  |  | Sig. (2-tailed) | .248 | .818 | .403 |
| *MT-ND4L* | | Correlation Coefficient | -.221 | -.074 | -.237 |
|  |  | Sig. (2-tailed) | .144 | .631 | .100 |
| *MT-ND4* | | Correlation Coefficient | -.028 | -.078 | -.040 |
|  |  | Sig. (2-tailed) | .854 | .616 | .782 |
| *MT-ND5* | | Correlation Coefficient | .065 | .055 | -.092 |
|  |  | Sig. (2-tailed) | .670 | .725 | .530 |
| *MT-ND6* | | Correlation Coefficient | .026 | .122 | -.102 |
|  |  | Sig. (2-tailed) | .864 | .429 | .485 |
| *MT-CYB* | | Correlation Coefficient | .006 | .118 | .234 |
|  |  | Sig. (2-tailed) | .967 | .445 | .105 |
| *CRHR1* | | Correlation Coefficient | 1.000 | ***.597*** | .304 |
|  |  | Sig. (2-tailed) | – | ***<.001*** | .042 |
| *CRHR2* | | Correlation Coefficient |  | 1.000 | .215 |
|  |  | Sig. (2-tailed) |  | – | .162 |
| *NR3C1* | | Correlation Coefficient |  |  | 1.000 |
|  |  | Sig. (2-tailed) |  |  | – |
| Strong (rho > 0.4) and significant (p < 0.05) non-parametric correlations between the expression of individual mitochondrial genes and the expression of *CRHR1*, *CRHR2* and *NR3C1* are reported in bold italicized. | | | | | |
