# Supplementary material for: Mitochondrial Gene Expression Profiles Are Associated with Maternal Psychosocial Stress in Pregnancy and Infant Temperament
Source: PLoS One. 2015 Sep 29;10(9):e0138929. doi: 10.1371/journal.pone.0138929 (PMC4587925; doi:10.1371/journal.pone.0138929)
Supplement: S6 Table — (DOCX) [file pone.0138929.s007.docx]

| Table S6. | Distribution of the MPSP individual indices and the MPSP summary score by Maternal Obsessive Compulsive Disorder (OCD). |
| --- | --- |
| \| **Group Statistics** \| \| \| \| \| \| \| --- \| --- \| --- \| --- \| --- \| --- \| \| Maternal OCD \| \| N \| Mean \| Std. Deviation \| Std. Error Mean \| \| Prenatal Perceived Stress \| No \| 84 \| 35.81 \| 7.102 \| .775 \| \| Yes \| 9 \| 40.89 \| 8.038 \| 2.679 \| \| State of Anxiety \| No \| 84 \| 37.37 \| 12.186 \| 1.330 \| \| Yes \| 9 \| 44.78 \| 9.922 \| 3.307 \| \| Trait of Anxiety \| No \| 84 \| 37.20 \| 10.431 \| 1.138 \| \| Yes \| 9 \| 45.11 \| 10.068 \| 3.356 \| \| Pregnancy Anxiety Total \| No \| 81 \| 5.8241 \| 2.13803 \| .23756 \| \| Yes \| 9 \| 8.0648 \| 2.23831 \| .74610 \| \| MPSP Summary Score \| No \| 81 \| -.1649801 \| .94920759 \| .10546751 \| \| Yes \| 9 \| .7395403 \| .93831601 \| .31277200 \|  \|  \| \| Sum of Squares \| Mean Square \| F \| Sig. \| \| --- \| --- \| --- \| --- \| --- \| --- \| \| Prenatal Perceived Stress \| Between Groups \| 209.804 \| 209.804 \| 4.060 \| .047 \| \| Within Groups \| 4702.942 \| 51.681 \|  \|  \| \| Total \| 4912.746 \|  \|  \|  \| \| State of Anxiety \| Between Groups \| 445.945 \| 445.945 \| 3.095 \| .049 \| \| Within Groups \| 13113.472 \| 144.104 \|  \|  \| \| Total \| 13559.417 \|  \|  \|  \| \| Trait of Anxiety \| Between Groups \| 508.213 \| 508.213 \| 4.699 \| .033 \| \| Within Groups \| 9841.135 \| 108.144 \|  \|  \| \| Total \| 10349.348 \|  \|  \|  \| \| Pregnancy Anxiety Total \| Between Groups \| 40.669 \| 40.669 \| 8.820 \| .004 \| \| Within Groups \| 405.775 \| 4.611 \|  \|  \| \| Total \| 446.444 \|  \|  \|  \| \| MPSP Summary Score \| Between Groups \| 6.627 \| 6.627 \| 7.371 \| .008 \| \| Within Groups \| 79.123 \| .899 \|  \|  \| \| Total \| 85.750 \|  \|  \|  \| | |
